# Indoor Finish Material Influence on Contamination, Transmission, and Eradication of Methicillin-Resistant *Staphylococcus aureus* (MRSA)

**DOI:** 10.1177/1937586720952892

**Published:** 2020-08-31

**Authors:** Debra Harris, Keyanna P. Taylor, Katie Napierkowski, Bernd Zechmann

**Affiliations:** 1 Human Sciences and Design, College of Health and Human Sciences, 14643 Baylor University, Waco, TX, USA; 2 College of Health and Human Sciences, 14643 Baylor University, Waco, TX, USA; 3 College of Arts and Sciences, 14643 Baylor University, Waco, TX, USA; 4 Center for Microscopy and Imaging, 14643 Baylor University, Waco, TX, USA

**Keywords:** environmental surface materials, finish materials, cleaning, disinfection, MRSA, pathogens, healthcare-associated infections

## Abstract

**Objective:**

The purpose of this study was to evaluate environmental surface materials used in healthcare environments for material composition, methicillin-resistant *Staphylococcus aureus* (MRSA) viability, and a comparison of two disinfectants, a bleach germicidal cleaner and Decon7, a novel disinfectant.

**Background:**

Contaminated environmental surfaces have been associated with outbreaks of healthcare-associated illness (HAIs). One in every 20 patients in U.S. acute care hospitals acquire a healthcare-associated illness, leading to consequences such as elevated morbidity, mortality, and a decrease in quality of life. In the patient environment, MRSA can remain viable from hours to up to 14 days.

**Methods:**

Environmental surface materials were evaluated as new and worn. Material composition and properties were assessed to evaluate surface integrity and the influence on the disinfection of MRSA. Inoculated materials were used to assess MRSA viability over time and the efficacy of a manufacturer’s recommended cleaning and disinfection product compared to a novel disinfectant.

**Results:**

Environmental surface materials respond differently in appearance and roughness, when mechanically worn. When measuring MRSA survival, at 24 hr, MRSA colony forming unit (CFU) counts were reduced on the copper sheet surface and solid surface with cupric oxide. By 72 hr, all MRSA counts were zero. Bleach and the novel disinfectant were equally effective at disinfecting MRSA from all surface types.

**Conclusions:**

This study highlights a gap in knowledge about the impact of type and wear of environmental surface materials used in healthcare environments on contamination with epidemiologically important organisms. In conclusion, environmental surface material wear, properties, and cleaning and disinfection efficacy are important factors to consider when addressing HAIs.

## Introduction and Background

Healthcare-associated infections (HAIs) are a leading cause of illness and death in the United States and worldwide (Han et al., 2015). Annually, an estimated 1.7 million patients suffer from HAIs in the United States, leading to about 100,000 deaths (Klevens et al., 2007). A recent study found that one of every 20 patients in U.S. acute care hospitals acquires an HAI, with the most prevalent pathogens being *Clostridium difficile* infection (CDI) and methicillin-resistant *Staphylococcus aureus* (MRSA; Klevens et al., 2007; Magill et al., 2014). Most HAIs are thought to be preventable; however, published mitigation guidelines are incongruent, obstructing a clear path for reduction success (Stone, 2009). Unresolved is the role of environmental surface contamination across the continuum of healthcare spaces as a contributing factor for HAIs.

During the last decade, substantial scientific evidence has increased, demonstrating that contamination of environmental surfaces in hospital rooms plays an important role in the transmission of several key healthcare-associated pathogens including MRSA, vancomycin-resistant Enterococcus, CDI, and norovirus (Boyce, 2007; Otter et al., 2013; Weber et al., 2013; Weber et al., 2010; Weinstein & Hota, 2004). One pathogen often linked in the literature to contaminated surfaces in hospitals is the gram-positive pathogen, MRSA. MRSA is a bacteria that is resistant to many antibiotics and may cause severe health problems including skin infections, fever, chest pain, fatigue, and muscle aches. Transmission of MRSA can occur directly from environment to patient or through healthcare workers, with one study showing that 42% of nurses contaminated their gloves by touching objects in the room of patients with MRSA without ever having touched the patient (Boyce et al., 1997). Furthermore, MRSA can remain viable for up to 14 days on surfaces and up to 9 weeks on cotton blanket material (Beard-Pegler et al., 1988; Duckworth & Jordens, 1990). The ability to survive on surfaces for an extended time is a clear justification that environmental surface materials play a significant role. This previous literature shows a clear justification for the claim that environmental surfaces in the patient environment should be given major consideration in preventing the transmission of MRSA.

***Furthermore, MRSA can remain viable for up to 14 days on surfaces and up to 9 weeks on cotton blanket material***.

### Purpose

The purpose of this study was to evaluate environmental surfaces used in healthcare environments for material influence in disinfection of MRSA, material composition, and compare a common disinfectant and Decon7 (Decon Seven Systems, Scottsdale, AZ), a biological disinfectant and chemical contaminant.

***The purpose of this study was to evaluate environmental surfaces used in healthcare environments for material influence in disinfection of MRSA, material composition, and compare a common disinfectant and Decon7***.

### Research Design

This experimental study sought to simulate contamination of new and worn environmental surface materials by patients or healthcare workers infected with MRSA through contact with surfaces and producing aerosols through coughing or sneezing. This study did not include human subjects or animals.

## Method

Five types of environmental surface samples were evaluated including stainless steel (SS), copper sheet (CS), high-pressure laminate (HPL), acrylic polymer solid surface (APS), and SCU with a total number of 150 environmental surface samples (Table 1).

**Table 1.** Environmental Surface Material Types, Including Stainless Steel, Acrylic Polymer Solid Surface, Solid Surface With Cupric Oxide, High-Pressure Laminate, and Copper Sheet.

Material	Color, Finish	Dimensions	Description
Acrylic polymer solid surface	White, no applied finish	4″ × 4″ × ½″ (101.6 mm × 101.6 mm × 12.7 mm)	Solid, nonporous, homogeneous, composed of acrylic resin and natural minerals
Solid surface with cupric oxide	Gray, no applied finish	3 ¾″ × 3 ¾″ × 3/8″ (95.25 mm × 95.25 mm × 9.53 mm)	Solid, homogeneous, antimicrobial sheet composed of polyester resins, mineral fillers, and pigments
Stainless steel, Grade 304	#4 finish brushed (annealed)	4″ × 4″ × 0.018″ (101.6 mm × 101.6 mm × 0.4572 mm)	Chromium-Nickel (CrNi) austenitic alloy sheet with 18% min. chromium and 10% max. nickel, 18 gauge
High-pressure laminate	White, matte finish	4″ × 4″ × 0.048″ (101.6 mm × 101.6 mm × 1.2192 mm)	Decorative surface papers impregnated with melamine resins pressed over kraft paper core sheets impregnated with phenolic resin
Copper sheet, antimicrobial	Natural copper, no applied finish	4″ × 4″ × 0.040″ (101.6 mm × 101.6 mm × 1.016 mm)	Copper alloy C71000 (Copper nickel, CuNi) composed of 78%–84% copper and 19.0%–23.0% nickel, 18 gauge

### Sample Preparation

Samples used as supplied were considered “new” representing environmental surface materials for initial use. The worn samples were abraded using the Taber Rotary Platform Abrasion Tester (Taber Industries, North Tonawanda, NY) to represent worn samples with a specific end point to simulate 6 years of normal wear for surface materials utilizing ASTM G195-18, modified for accelerated wear.

Material documentation was assessed to determine the manufacturer’s intended useful life. Failure tests were conducted on high-pressure plastic laminate to set a baseline. Visual assessment for wear was reached, indicated by wear through of printed pattern/color in all four quadrants of the sample was used to determine failure (American National Standards Institute, 2005). The first test utilized the CS-10 wheel and the material failed at 2,500 cycles, the second test utilized the CS-17 wheel and the material failed at 3,945 cycles, and the final test utilized the S-35 wheel and the material failed at 500 cycles. All tests used a wheel loading of 1,000 g and a vacuum suction level of 60 (100%) with a height of the vacuum pick up nozzle set to 1.5 mm (0.059 in.) above the sample surface. Based on these tests, it was determined that the S-35 wheel was to be used on all materials at 0.60 cycles (300) to simulate 6 years of accelerated use across all material types (ANSI, 2005).

***Based on these tests, it was determined that the S-35 wheel was to be used on all materials at 0.60 cycles (300) to simulate 6 years of accelerated use across all material types (ANSI, 2005)***.

### Environmental Surface Material Testing

With the accelerated wear test established, 60 samples were abraded in ambient room conditions (temperature at 69°F [20.56°C]; humidity at 48%). Each sample was weighed before and after the abrasion test and analyzed for mass loss.

The data were analyzed for percent mass loss by type of material and within the type of material comparing new and worn. The data were transformed to satisfy assumptions of normality and homogeneity of variances. Analysis of variance was conducted on the transformed data to determine whether there was a difference in the five environmental surface materials. Since there was a difference in the means of the transformed variables (*p* < .0001), post hoc multiple comparisons were conducted. Tukey’s honestly significant difference test for pairwise comparisons was used to compare new and worn within material type and compare for mass loss by type of material.

Scanning electron microscopy (SEM) and atomic force microscopy (AFM) were used for surface characterization of new and worn samples. For SEM, small samples of the different surfaces (1 cm^2^; 0.155 in.) were mounted on aluminum mounts with carbon tape. Samples were then imaged with a Hitachi TM3030 Plus tabletop SEM (Hitachi High Technologies America, Irving, TX) in low vacuum mode, with 15 kV, and 10 mm (0.397 in.) working distance. For AFM studies, the same samples were mounted on metal discs with super glue and imaged with a Dimension Icon AFM (Bruker Corporation, Billerica, MA) in peak force tapping mode. Surface roughness was analyzed with the software Nanoscope Version 9.4.

### Microbiological Specimen Preparation

The initial bacterium received was a freeze-dried pellet of *Staphylococcus aureus* subspecies aureus Rosenbach. To prepare the initial bacterium into a suspension for inoculation, serial dilutions were completed using a smear plate method and optical densities were recorded using a Thermo Electron Spectronic 20D+ Single Beam Spectrophotometer (Thermo Fisher, Waltham, MA). The serial dilutions and recorded optical densities were used to determine the bacteria’s growth rate and calculate suspension concentration. Utilizing the same method for serial dilutions, the suspension used to inoculate the surface material samples had a concentration of 1.375 × 10^5^ CFU/ml.

### Microbiological Testing

Coded and sterilized samples (60), triplicates of new materials, were inoculated with a prepared MRSA suspension equivalent to 1.375 × 10^5^ CFU/ml. Samples were inoculated with, thirty 100 µl (or .1 ml) droplets of the prepared suspension were placed into each sample. This size droplet was used to better simulate small droplets of contaminated body fluids from colonized patients and to ensure that the suspension would dry on each surface. This drop size and number of drops vary in comparison to Lankford et al. (2006) who used three hanging drops of approximately 250 µl (.25 ml) to inoculate surfaces.

To determine the length of time MRSA survives on environmental surface materials in a controlled environment, samples were inoculated and stored covered with sterile petri dishes in an incubator at 35°C (95°F) and cultured by tryptic soy agar contact plates at the designated time points. The time points measured were 5 min, 2 hr, 24 hr, and 72 hr.

Evaluating the effectiveness of manufacturer’s recommended cleaning and disinfecting processes for environmental surface materials in healthcare facilities and laboratories required inoculation of 30 samples (triplicates) that were then stored uncovered in a Sterigard III Advance model, Class II Type A/B3 hood (The Baker Company, Sanford, ME) for approximately 2 hr. Two hours were chosen to ensure that surfaces would dry before being contact plated. Once surfaces were dry, they were contact plated and then cleaned and disinfected by spraying and air drying in the fume hood with a minimum contact time of 10 min. The chemical disinfectant used to assess recommended cleaning and disinfection was Clorox Healthcare Bleach Germicidal Cleaner (The Clorox Company, Oakland, CA). These procedures were replicated with another set of 30 samples, triplicates of new and worn samples for the 5 materials, to assess the effectiveness of Decon7, a disinfectant and decontaminant developed for bio and chemical warfare, using recommended cleaning and disinfecting processes for environmental surface materials in healthcare facilities and laboratories. The conditions in the fume hood were recorded as at 25.55°C (78°F) and 49% relative humidity and 26.11°C (79°F) and 44% relative humidity for the healthcare germicidal bleach and Decon7, respectively.

For each test, after incubation, plates were counted and recorded. For statistical analysis related to the length of time MRSA survives on the environmental surface materials, the CFU measurements were treated as ordinal categories, including too few to count (TFTC) and too many to count (TMTC). The ordinal categories were 0, TFTC, a numerical count for 30–300, and TMTC. These categories were collapsed into treatments of three groups (CS and SCU, APS and HPL, and SS) to determine whether the category was dependent on material type at each time (5 min, 2 hr, 24 hr, and 72 hr) using Fisher’s exact test.

## Results

### Environmental Surface Materials

#### Percent mass loss by type of environmental surface material

**Figure 1.**
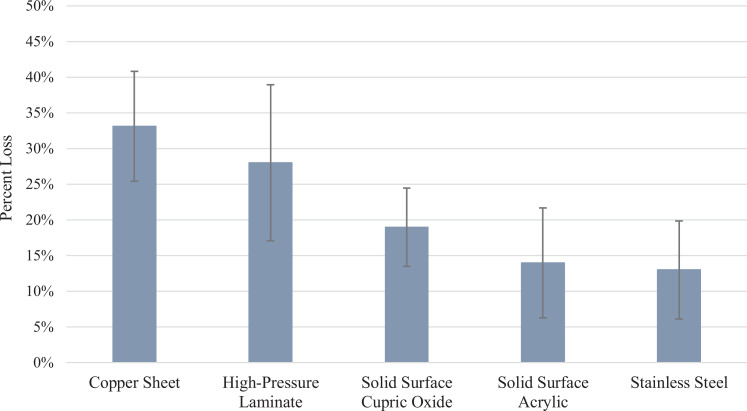
Mean mass percent loss for five environmental surface materials after simulated 6 years use. *Note*. Standard deviations are represented by error bars for each set of triplicate surface samples.

There was an interaction between material type and wear, but only for CS (*p* < .0001). There were statistically significant differences between CS and the APS (*p* = .0006), between CS and the SCU (*p* = .0151), and between CS and SS sheet (*p* = .0002). Significant differences were also found between HPL and the APS (*p* = .0245) and SS (*p* = .01). Multiple comparisons showed no difference in percentage mass loss between SS, APS, or the SCU. No difference was shown between the SCU and HPL nor between HPL and CS. CS had the most percent mass loss (33%) and the SS had the least percent mass loss (13%; Figure 1).

#### Percent mass loss of new and aged samples by environmental surface material type

When comparing new and worn surfaces of the same material type, all materials except SS indicated a significant difference: (1) CS (*p* = .0001), (2) APS (*p* = .0353), (3) SCU (*p* = .0009), and (4) HPL (*p* = .0001).

#### Microscopy

**Figure 2.**
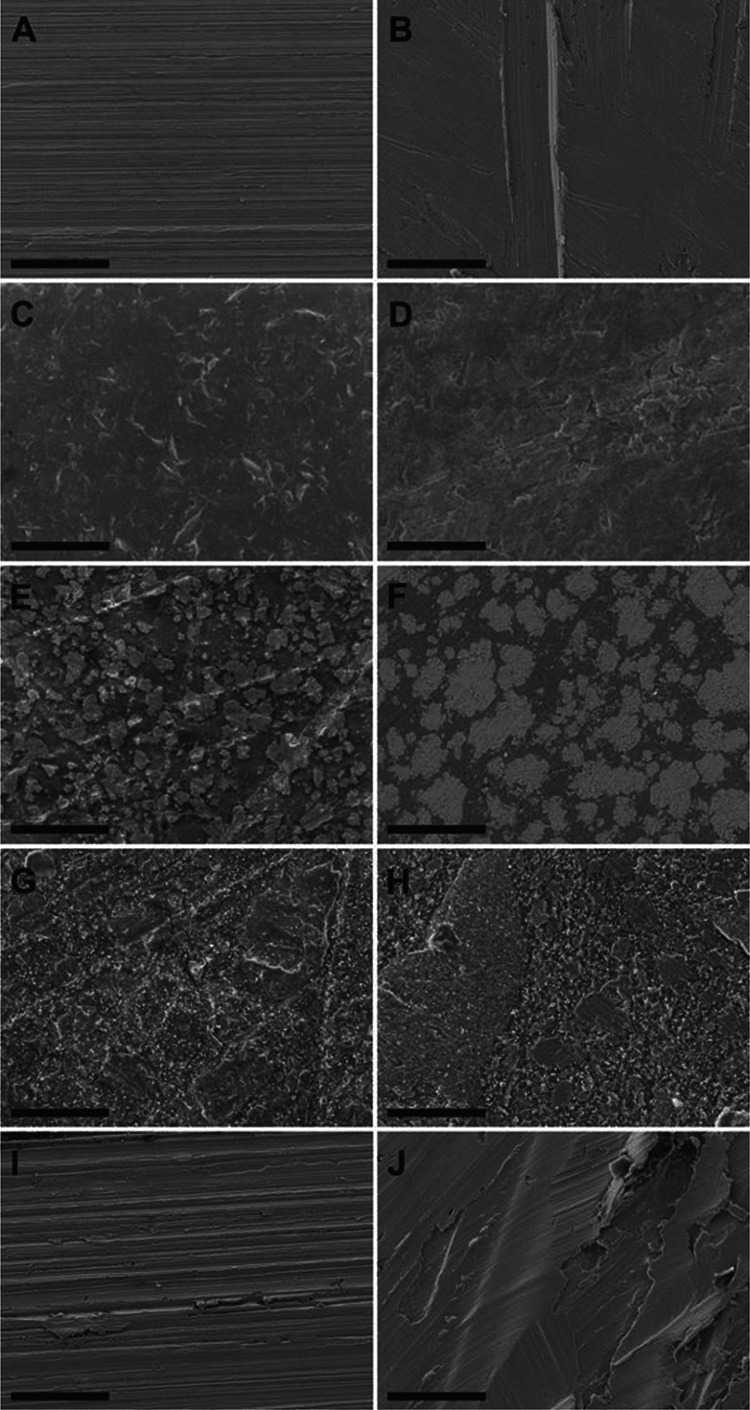
Scanning electron micrographs of new (A, C, E, G, I) and worn (B, D, F, H, J) samples of each environmental surface type. Samples represent stainless steel (A, B), acrylic polymer solid surface (C, D), high-pressure laminate (E, F), copper sheet (H, H), and solid surface with cupric oxide (I, J). Bar = 100 μm.

**Figure 3.**
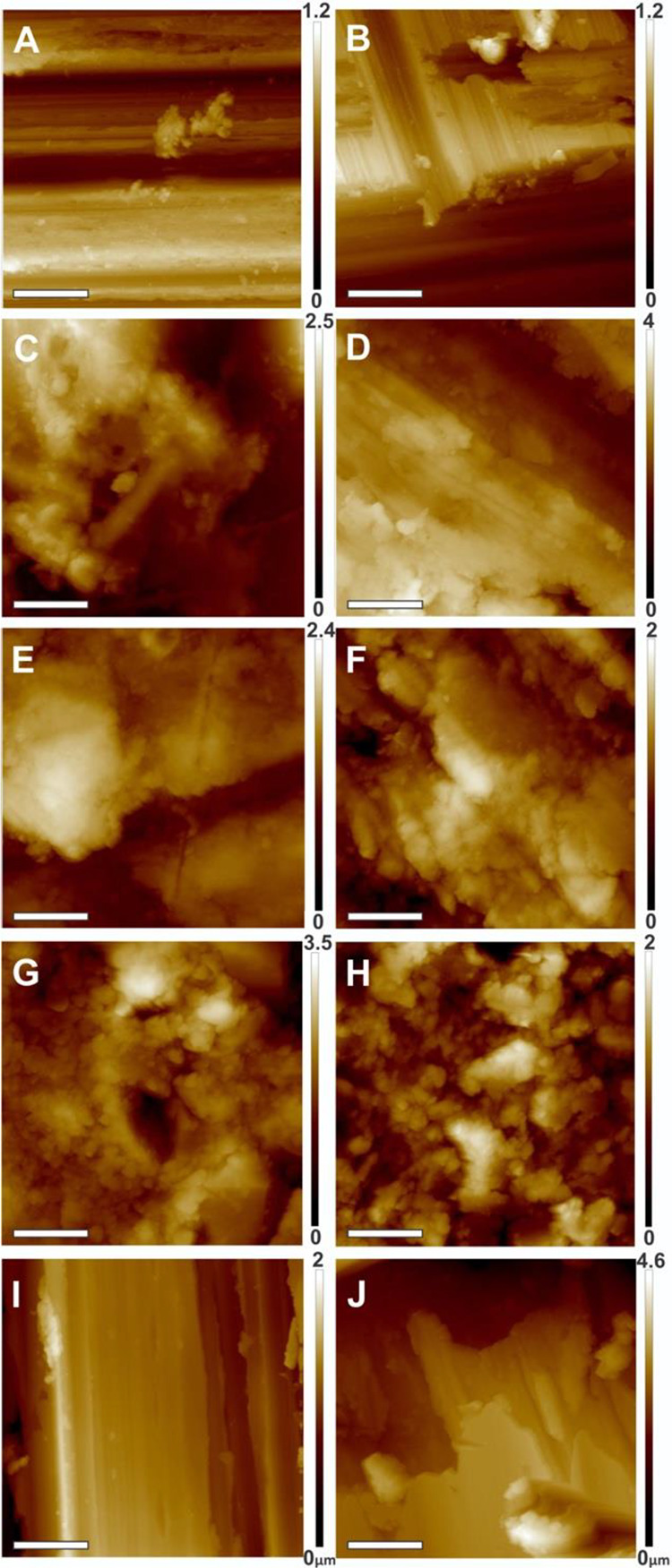
Atomic force micrographs of new (A, C, E, G, I) and worn (B, D, F, H, J) samples of each environmental surface type. Images represent stainless steel (A, B), acrylic polymer solid surface (C, D), high-pressure laminate (E, F), copper sheet (H, H), and solid surface with cupric oxide (I, J). Bar = 5 μm.

**Figure 4.**
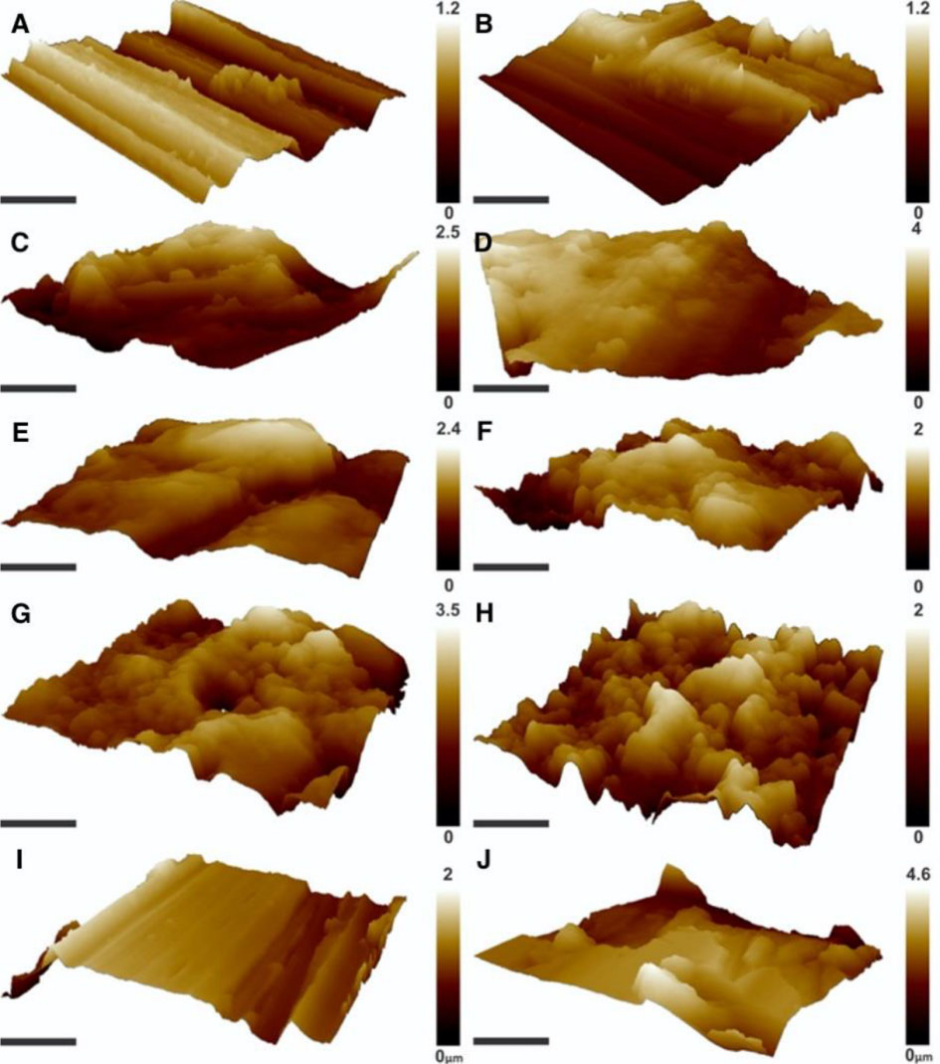
Atomic force micrographs of the three-dimensional surface topography of new (A, C, E, G, I) and worn (B, D, F, H, J) samples of each environmental surface type. Images represent stainless steel (A, B), acrylic polymer solid surface (C, D), high-pressure laminate (E, F), copper sheet (H, H), and solid surface with cupric oxide (I, J). Bar = 5 μm.

SEM and AFM of new and worn samples revealed differences in surface topography among the different samples (Figures 2 and 3). While new SS showed the least surface roughness of all analyzed surfaces (113 nm), new APS and HPL showed the highest surface roughness (238 and 236 nm). While the worn surface of SS and SCU appeared smooth in the SEM and AFM (Figures 2 and 3—A, B, I, J), the surface of APS, HPL, and CS appeared flaky (Figures 2 and 3—X, C, D, E, F, G, H). The wearing of the material had different effects on the surface topography of the different materials (Figure 4). Wearing strongly increased surface roughness of CS samples (71.6%) but decreased surface roughness of SCU (47.8%) and APS (9.7%; Table 2).

**Table 2.** Surface Roughness Analyzed by Atomic Force Microscopy.

Sample Type	Surface Roughness (nm)		New to Worn Surface Roughness Change (%)
	New	Worn	
Acrylic polymer solid surface (APS)	238	215	−9.7
Solid surface with cupric oxide (SCU)	143	74.7	−47.8
Stainless steel (SS), Grade 304	113	100	−11.5
High-pressure laminate (HPL)	236	240	+1.7
Copper sheet (CS), antimicrobial	183	314	+71.6

### Microbiological Performance

When assessing the length of time MRSA survives on the environmental surface samples, GMRSA survived between 2 and 24 hr, while for the APS and SS, MRSA expired between 24 and 72 hr. At 5 min, all surfaces besides SCU showed too many CFUs to count (>300). At 2 hr, every surface type showed too many CFUs to count. At 24 hr, CS, SCU, and HPL showed no CFUs, while the APS and SS surface samples showed low numbers (ranging from 52 to 147) of MRSA CFUs still present. Lastly, at 72 hr, all surfaces showed no CFUs remaining.

Environmental surface materials were evaluated for dependence of type of material. At 5 min and two hr, all material types measured TMTC, indicating that CFU counts were independent of material type. At 24 hr, there was an association between material type and CFU counts (*p* = .0156). The combined copper category had all CFU measurements of 0 at this time, while one of the plastics and SS had CFU measurements in the countable range (30–300). At 72 hr, the CFU count was independent of the material type.

When comparing CS and SCU at each time point, at 5 min, CS was more likely to have TMTC CFUs than the SCU. At 2 hr, both materials had TMTC CFUs. At 24 hr, both types of materials had no CFUs with no significant differences.

The effectiveness of a manufacturer’s recommended cleaning and disinfecting process was tested using healthcare bleach germicidal cleaner on all inoculated surface material types, with results showing that MRSA was eradicated. Similarly, to the healthcare bleach germicidal cleaner, the effectiveness of Decon7 was tested, with results showing that all surface types inoculated with MRSA were eradicated. However, after inoculation and before cleaning and disinfection, CS and SCU showed lower CFUs of MRSA compared to all other surfaces.

## Discussion

The purpose of this study was to evaluate a selection of environmental surface materials used in healthcare environments to determine differences in material composition and influence on the viability of MRSA; and compare a common disinfectant, healthcare bleach germicidal cleaner to Decon7, an EPA registered biological disinfectant for norovirus.

In this Study, all surfaces of the materials performed differently when worn except for SS. SS is a hard metal and did not lose significant mass, nor change surface characteristics after wearing. All other materials lost significant mass after the wear study. When comparing materials, CS and HPL significantly lost mass compared to SS and the two solid surfaces.

***In this Study, all surfaces of the materials performed differently when worn except for SS.***.

Microscopy of the new and worn environmental surface materials revealed differences in surface topography among the different materials. Of the new materials, SS (brushed) and HPL appeared to have the smoothest surface, while the APS and HPL appeared to have the roughest surface. Of the worn materials, the surfaces of the SS and the SCU were smooth, compared to the APS, HPL, and CS, which appeared flaky. These results suggest that the surface of the SS did not significantly change; however, after wear, the SCU became smoother, while the remaining surface materials increased in roughness. Future research should investigate if topographical changes increase or decrease the risk of forming biofilms or providing reservoirs for pathogens. Cleaning efficacy is influenced by the surface contamination, properties of each surface material, the cleaning and disinfection technology used, and the efficiency of environmental services staff. Environmental surfaces with differing material properties may be a factor that should be considered in specification with concern for cleaning and disinfection of patient environments where HAIs are common.

MRSA survival time from undisturbed surfaces in a controlled environment was between 24 and 72 hr. However, a reduction in MRSA CFUs was found on the CS and solid surface infused with cupric oxide between 2 and 24 hr after inoculation. Previous studies echo similar results that focus on the use of copper in surface materials as a natural antimicrobial agent that degrades cell structure and reduces both gram-negative and gram-positive bacteria (Eser et al., 2015; Esolen et al., 2018; Jing et al., 2018; Schmidt et al., 2017). Sharpe and Schmidt (2011) found that antimicrobial copper alloy products reduce the bioburden over time and that the bioburden was reduced on adjacent materials, suggesting a potential microbiological halo effect, which should be studied further. Additional studies with similar results indicated the efficacy of copper as an antimicrobial surface for HAIs such as MRSA (Humphreys, 2013; Schmidt et al., 2012, 2016). To be registered with the Environmental Protection Agency (EPA, 2016) as an antimicrobial product and to use the claim for “continuous reduction” of bacterial, antimicrobial copper alloy products must achieve a 3 log_10_ reduction in viable bacteria within a 1-hr contact time.

***…a reduction in MRSA CFUs was found on the CS and solid surface infused with cupric oxide between 2 and 24 hr after inoculation***.

Healthcare bleach germicidal cleaner and Decon7 were effective in disinfecting MRSA on all surface types. There was no interaction between material type and disinfection. The Centers for Disease Control and Prevention have established guidelines for cleaning and disinfection in healthcare settings, which recommend cleaning and disinfecting noncritical environmental surfaces with EPA registered low- or intermediate-level disinfectants (Rutala et al., 2008). However, our study utilized Decon7, a biological and chemical decontaminant developed for biowarfare to eradicate biological contaminants such as antibiotic-resistant *Escherichia coli*, antibiotic-resistant *Klebsiella pneumoniae*, *Clostridium difficile*, human coronavirus, and SARS-CoV-1. Future studies should test for efficacy and material compatibility.

***Healthcare bleach germicidal cleaner and Decon7 were effective in disinfecting MRSA on all surface types***.

### Limitations

This study had several limitations to consider. When conducting the wear study, a modified standard was used to develop a protocol for accelerated wear testing. This study only tested for mechanical wear, but future studies should utilize methods for accelerated life testing that include wear, heat, ultraviolet (UV) light, humidity, and chemical erosion. MRSA time points for survival should be selected for higher sensitivity. The crucial time frame for tracking the growth of MRSA in a controlled environment was between 5 min and 24 hr. Reducing the inoculum to decrease MRSA CFUs would be beneficial as well as counting all CFUs so that the data would be numerical and not categorical.

### Recommendations for Future Research

Future research should aim to develop a validated methodology to conduct accelerated life testing of environmental surfaces. A methodology for testing accelerated life of environmental surfaces should be comprehensive including more factors that may elucidate limits of the materials and potential incompatibilities such as chemical exposure, temperature, humidity, and UV light. Future research should continue to evaluate how long pathogens are viable on surface materials to determine significant differences that may inform specifications for the built environment. Observing the prevalence of MRSA on surfaces between 2 hr and 24 hr postinoculation is a key time frame to discover the specific lifetime of MRSA on various environmental surfaces. Future studies should examine the role of environmental surface materials on pathogen survival, and cleaning and disinfection efficacy in an applied healthcare environment. This study was conducted in a controlled laboratory environment without soil loads. Other research has shown that MRSA can live on surfaces up to 14 days and cotton blankets for up to 9 weeks. Future studies should consider expanding the experiments to use soil and to conduct field experiments where MRSA survival and cleaning protocols may vary. Finally, future studies should utilize an expanded selection of environmental surface materials, pathogens, and cleaning methods.

## Conclusion

The results of this study suggest that environmental surfaces behave differently when worn; therefore, a careful selection of materials should be considered in high use environments like healthcare. Antimicrobial materials, and cleaning and disinfection methods and technologies should be studied further in the context of pathogen contamination and transmission in healthcare environments. While chemical disinfection and cleaning procedures in the healthcare environment have been studied extensively, this study highlights a gap in knowledge regarding the impact of type and wear of environmental surface materials used in healthcare environments on pathogens with epidemiologically important organisms. In conclusion, environmental surface material wear, properties, and cleaning and disinfection efficacy are important factors to consider when addressing HAIs.

## Implications for Practice

- Environmental surface material wear, properties, and cleaning and disinfection efficacy are important factors to consider when addressing the specification of materials for healthcare environments.
- The impact of wear on environmental surface material properties may provide a reservoir for pathogens.
- Copper and copper-infused surfaces reduced the bioburden, suggesting a continuous reduction that may contribute to a productive reduction in overall contamination.
- Cleaning and disinfection methods such as novel disinfectants should be considered in the context of pathogen contamination and transmission in healthcare environments.
